# The Role of the Subthalamic Nucleus in Sequential Working Memory in *De Novo* Parkinson's Disease

**DOI:** 10.1002/mds.28344

**Published:** 2020-10-24

**Authors:** Zheng Ye, Guanyu Zhang, Yingshuang Zhang, Shuaiqi Li, Na Liu, Xiaolin Zhou, Weizhong Xiao, Thomas F. Münte

**Affiliations:** ^1^ Institute of Neuroscience, Key Laboratory of Primate Neurobiology, Center for Excellence in Brain Science and Intelligence Technology Chinese Academy of Sciences Shanghai China; ^2^ Institute of Psychology Chinese Academy of Sciences Beijing China; ^3^ Department of Psychology University of Chinese Academy of Sciences Beijing China; ^4^ Department of Neurology Peking University Third Hospital Beijing China; ^5^ Center for Brain and Cognitive Sciences School of Psychological and Cognitive Sciences, Peking University Beijing China; ^6^ PKU‐IDG/McGovern Institute for Brain Research Peking University Beijing China; ^7^ Department of Neurology University of Lübeck Lübeck Germany

**Keywords:** fMRI; Parkinson's disease; sequential working memory; subthalamic nucleus

## Abstract

**Background:**

Deficits in maintaining and manipulating sequential information online can occur even in patients with mild Parkinson's disease. The subthalamic nucleus may play a modulatory role in the neural system for sequential working memory, which also includes the lateral prefrontal cortex.

**Objectives:**

The objective of this study was to investigate neural markers of sequential working memory deficits in patients with de novo Parkinson's disease.

**Methods:**

A total of 50 patients with de novo Parkinson's disease and 50 healthy controls completed a digit ordering task during functional magnetic resonance imaging scanning. The task separated the maintenance (“pure recall”) and manipulation of sequences (“reorder & recall” vs “pure recall”).

**Results:**

In healthy controls, individual participants' task accuracy was predicted by the regional activation and functional connectivity of the subthalamic nucleus. Healthy participants who showed lower subthalamic nucleus activation and stronger subthalamic nucleus connectivity with the putamen performed more accurately in maintaining sequences (“pure recall”). Healthy participants who showed greater ordering‐related subthalamic nucleus activation change exhibited smaller accuracy costs in manipulating sequences (“reorder & recall” vs “pure recall”). Patients performed less accurately than healthy controls, especially in “reorder & recall” trials, accompanied by an overactivation in the subthalamic nucleus and a loss of synchrony between the subthalamic nucleus and putamen. Individual patients' task accuracy was predicted only by the subthalamic nucleus connectivity. The contribution of the subthalamic nucleus activation or activation change was absent. We observed no change in the lateral prefrontal cortex.

**Conclusions:**

The overactivation and weakened functional connectivity of the subthalamic nucleus are the neural markers of sequential working memory deficits in de novo Parkinson's disease. © 2020 The Authors. *Movement Disorders* published by Wiley Periodicals LLC on behalf of International Parkinson and Movement Disorder Society

When we schedule our day, we may arrange tasks in the order they come or prioritize a task that is due first. A critical ability involved in this scenario is the ability to maintain and manipulate sequential information online. This ability is sophisticated in humans and chimpanzees^1^ but vulnerable to neurodegenerative diseases. In Parkinson's disease (PD), deficits in sequential working memory can occur even in patients with mild clinical symptoms,^2^, ^3^ which potentially lead to difficulties in planning sequential steps to solve problems and understanding temporal relations of events expressed out of chronological order.^4^, ^5^, ^6^, ^7^ In this study, we aimed to investigate neural markers of the deficits in de novo patients with mild PD using functional magnetic resonance imaging (fMRI). The use of a newly diagnosed and unmedicated cohort allows us to separate the contribution of the disease from that of chronic medication.

The cognitive and neural mechanisms that code and retrieve sequential information may differ from the mechanisms that code and retrieve item‐specific information (eg, color).^8^, ^9^, ^10^ Recently we described a neural system for sequential working memory comprising the lateral prefrontal cortex, posterior parietal cortex, subthalamic nucleus (STN), globus pallidus, and thalamus. The lateral prefrontal and posterior parietal regions were more activated and more strongly connected with the supplementary motor area when healthy adults processed sequential information. The effect of age exhibited as a widely spreading overactivation in the prefrontal and parietal regions and a weakened psychophysiological interaction between the prefrontal/parietal regions and supplementary motor area.^11^

Cognitive decline in PD correlates with the spread of misfolded α‐synuclein from the brainstem to limbic and neocortical structures.^12^ The etiology of PD‐related decline may differ from that of age‐related decline. In this study, we examined how the disease compromises the neural system for sequential working memory and alters behavioral performance in de novo patients with mild PD. To capture the neural processes of sequential working memory, we combined a digit ordering task (Fig. 1) with fMRI. In each trial, participants had to remember a sequence of 4 different digits in ascending order. In “pure recall” trials, the digits were presented already in ascending order, and there was no need for reordering. In “reorder & recall” trials, the digits were fully randomized, and participants always had to reorder them to generate a new sequence. The “pure recall” trials measured the temporary maintenance of sequences (including encoding, storage, and retrieval), whereas the contrast of “reorder & recall” versus “pure recall” trials emphasized the flexible manipulation of sequences. First, we sought to replicate the neural system for sequential working memory as in healthy adults.^11^ Second, we wanted to examine whether patients with PD had weaker regional activation or functional connectivity in the basal ganglia or lateral prefrontal cortex. Third, we aimed to determine whether observed changes in regional activation or functional connectivity can predict the performance of sequence maintenance or manipulation.

**FIG. 1:**
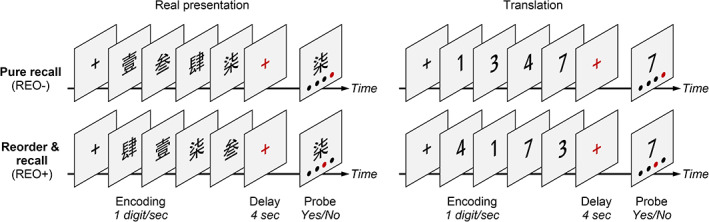
Digit ordering task. The task included interleaved “pure recall” (REO−, 30 trials) and “reorder & recall” trials (REO+, 32 trials). In each trial, participants read a sequence of 4 different digits written in Chinese (1 digit/s). They had to remember the digits in ascending order through a short delay (4 seconds). In “pure recall” trials, the digits were presented in ascending order. In “reorder & recall” trials, the digits were randomized, and participants always had to reorder them. After the delay, participants saw a digit probe with 4 dots indicating 4 positions from left to right. They had to judge whether the red dot indicated the target position of the digit probe by pressing buttons with the right hand. [Color figure can be viewed at wileyonlinelibrary.com]

## Methods

This study was approved by the ethics committee of the Peking University Third Hospital according to the Declaration of Helsinki. Each participant signed a written informed consent before participating in this study.

### Patients and Clinical Assessment

We recruited 50 patients with idiopathic PD (Movement Disorder Society [MDS] Clinical Diagnostic Criteria for Parkinson's Disease^13^) at the Peking University Third Hospital Department of Neurology between 2018 and 2020. Inclusion criteria were (1) newly diagnosed PD, (2) having not been treated with antiparkinsonian drugs, (3) Hoehn and Yahr Stages 1 to 2.5, (4) age 40 to 75 years, (5) education ≥9 years, (6) Mandarin Chinese speaking, and (7) right‐handed. Exclusion criteria were (1) diagnosed with atypical parkinsonism; (2) a history of epilepsy, stroke, or brain injury; (3) alcohol or drug abuse; (4) past significant psychiatric disorders or intake of antipsychotic drugs in past 3 months; (5) possible current depression (Beck Depression Inventory II >7) or intake of antidepressants in past 3 months; (6) possible dementia (Montreal Cognitive Assessment <21/30) or intake of antidementia drugs in past 3 months; (7) working memory spans (Adaptive Digit Ordering Test,^14^ Digit Span Forward Test) <4; and (8) contraindications to MRI. We assessed all patients with the Movement Disorder Society–sponsored revision of the Unified Parkinson's Disease Rating Scale (MDS‐UPDRS) Parts I and III subscales. We identified patients' motor subtypes using their MDS‐UPDRS Part III scores.^15^ Table 1 shows the demographic, clinical, and neuropsychological data of the patients and healthy controls.

**TABLE 1:** Demographic, clinical, and neuropsychological data of patients with PD and healthy controls (means, standard deviations, and group differences)

Features/measures	De Novo PD, N = 50	Healthy control, N = 50	Group differences, *P* values
Male/female	25/25	25/25	1.00
Age, y	58.6 (9.0)	57.7 (5.5)	0.54
Education, y	13.1 (2.9)	13.0 (2.0)	0.87
Motor symptoms			
Hoehn and Yahr	1.9 (0.5)	—	—
MDS‐UPDRS Part III: motor examination	15.5 (7.7)	—	—
Subtype: tremor‐dominant/akinetic‐rigid/mixed	5/25/20	—	—
Family history of tremor, Y/N	11/39	—	—
Cognition			
Montreal Cognitive Assessment	26.7 (2.0)	28.1 (1.3)	<0.001*
Digit Span Forward	7.9 (1.3)	7.8 (0.9)	0.66
Adaptive Digit Ordering	6.1 (1.8)	7.5 (1.9)	0.001*
Animal fluency	18.4 (3.9)	20.5 (4.8)	0.02
Other nonmotor functions			
MDS‐UPDRS Part I: nonmotor experiences of daily living	5.1 (3.0)	—	—
Hyposmia, Y/N	10/40	—	—
Constipation, Y/N	20/30	—	—
Beck Depression Inventory II	3.1 (2.4)	1.5 (1.4)	<0.001*
REM Sleep Behavior Disorder Screening Questionnaire	3.5 (1.9)	1.7 (1.5)	<0.001*
Epworth Sleep Scale	3.8 (4.0)	3.3 (2.4)	0.47

Group differences, *P* values of 2‐sample *t* tests, or Kruskal–Wallis test as appropriate. *Significant differences thresholded at *P* < 0.005 (Bonferroni correction for 10 tests).

PD, Parkinson's disease; MDS‐UPDRS, Movement Disorder Society–sponsored revision of Unified Parkinson's Disease Rating Scale; REM, rapid eye movement.

### Healthy Control Subjects

We recruited 50 age‐matched and education‐matched healthy controls. Exclusion criteria were (1) a history of significant neurological or psychiatric disorders, (2) alcohol or drug abuse, (3) possible current depression, (4) possible dementia or mild cognitive impairment (Montreal Cognitive Assessment <26/30), and (5) working memory spans <4.

### Experimental Procedure

All participants completed the digit ordering task (Fig. 1), including a practice block before scanning (4 minutes) and 2 experimental blocks during scanning (7–8 minutes each). We measured all participants between 8 and 10 am of the day to minimize the potential effects of circadian variations in the striatal dopamine release.^16^, ^17^

### Statistical Analysis of Behavioral Data

We controlled the quality of behavioral data by monitoring premature (trials with a reaction time shorter than 0.1 second) and inattentive responses (trials with a reaction time that was 3 standard deviations above the mean). Participants made no premature responses and very few inattentive responses (∼1%).

First, we examined whether patients with PD responded less accurately (percentage of correct trials) or more slowly (mean reaction time of correct trials) than healthy controls using repeated‐measures analyses of variance (ANOVAs) (1‐tailed, *P* < 0.025 for Bonferroni correction). The ANOVA had a within‐subject factor trial type (“reorder & recall,” “pure recall”), and a between‐subject factor group (PD, healthy control). Second, we examined whether individual patients' task accuracy (arcsine transformed^18^) correlated with their severity of nonmotor (MDS‐UPDRS Part I score) or motor symptoms (MDS‐UPDRS Part III score) (2‐tailed, *P* < 0.025 for Bonferroni correction).

### MRI Acquisition and Preprocessing

Brain imaging data were acquired on a General Electric Discovery MR750 3.0T scanner with an 8‐channel head coil. High‐resolution T1‐weighted images used an inversion recovery prepped‐fast spoiled gradient recalled echo imaging sequence (192 sequential sagittal slices, 450‐millisecond time of inversion, 7‐millisecond time of echo, 12° flip angle, 256 × 256 mm^2^ field of view, 1‐mm thickness, no gap, and 1 × 1 mm^2^ in‐plane resolution). Functional T2‐weighted images used a standard echo‐planar imaging sequence (33 interleaved ascending axial slices, 2000‐ millisecond time of repetition, 30‐ millisecond time of echo, 90° flip angle, 224 × 224 mm^2^ field of view, 4.2‐mm thickness, no gap, and 3.5 × 3.5 mm^2^ in‐plane resolution).

fMRI data were preprocessed using SPM12 (revision 7219, www.fil.ion.ucl.ac.uk/spm). The first 5 images of each experimental block were discarded. Other images were realigned to a mean functional image, corrected for slice acquisition time difference, registered to the high‐resolution T1‐weighted image, normalized to the Montreal Neurological Institute and Hospital coordinate system,^19^ resampled to voxels of 3 × 3 × 3 mm^3^, smoothed with a Gaussian kernel of 6‐mm full‐width half‐maximum, and filtered with a 128‐second high‐pass filter.

We controlled the quality of fMRI data preprocessing. A total of 5 participants from each group who had excessive head motion (total displacement >3 mm) or suboptimal spatial normalization (visual inspection) were excluded from fMRI data analysis. In the included participants, patients with PD did not move more than healthy controls.

### Statistical Analysis of fMRI Data

First, we replicated the ordering‐related regional activation and deactivation.^11^ At the subject level, the general linear model convolved a design matrix with a canonical hemodynamic response function. The design matrix included correct and incorrect “reorder & recall” trials and “pure recall” trials as separate regressors. A comprehensive indicator of head motion was derived from estimated motion parameters and included as a nuisance regressor.^20^ Each trial was time locked to its onset and modeled with its real duration. Classical parameter estimation was applied with a 1‐lag autoregressive model. The ordering‐related activation was defined as “reorder & recall” versus “pure recall” and ordering‐related deactivation as “pure recall” versus “reorder & recall.” At the group level, we conducted whole‐brain 2‐sample *t* tests (voxel‐level *P* < 0.05 family‐wise‐error corrected).

Second, we detected group differences in regional activation of the STN, globus pallidus, lateral prefrontal regions (BA46/9, BA44/45), and default mode network regions (medial prefrontal cortex, posterior cingulate cortex). To create task‐specific and anatomically precise masks, we obtained the contrast map of “reorder & recall” versus “pure recall” from an independent fMRI data set (24 healthy adults)^11^ and overlapped it with the Basal Ganglia Human Area Template,^21^ Brodmann area,^22^ or default mode network templates,^23^ generating 6 regions of interest. For each region of interest, we extracted the percent signal change relative to the whole‐brain mean signal and entered it into a repeated‐measures ANOVA (2‐tailed, *P* < 0.05). The ANOVA had a within‐subject factor trial type (“reorder & recall,” “pure recall”) and a between‐subject factor group (PD, healthy control).

Having observed group differences in regional activation of the STN and globus pallidus, third, we detected group differences in their functional connectivity (correlations between physiological signals) and psychophysiological interaction (PPI). Because of its consistent involvement in working memory processes, we included the left dorsolateral prefrontal cortex as a third seed although it showed no group difference in regional activation. The time course of each seed was extracted, demeaned, and deconvolved to create the PPI variable. At the subject level, the general linear model included a physiological signal regressor, a PPI regressor, and a psychological contrast regressor (“reorder & recall” vs “pure recall”).^24^ The estimated parameter of the physiological signal regressor indicated the degree to which the time course of a voxel correlated with the time course of the seed. The estimated parameter of the PPI regressor indicated the degree to which the functional connectivity between the seed and the voxel was modulated by trial type. At the group level, we conducted whole‐brain 2‐sample *t* tests (voxel‐level *P* < 0.001, cluster‐level *P* < 0.05, family‐wise‐error corrected).

Fourth, we examined whether individual differences in task accuracy can be predicted by the regional activation or functional connectivity of the STN using linear regression models (2‐tailed, *P* < 0.025 for Bonferroni correction). The first model tested whether the “pure recall” accuracy can be predicted by the “pure recall” STN activation or the STN functional connectivity with the putamen or posterior cingulate cortex. The second model tested whether the ordering‐related accuracy change (“reorder & recall” vs “pure recall”) can be predicted by the corresponding STN activation change or the STN functional connectivity with the putamen or posterior cingulate cortex. The accuracy and connectivity values were arcsine transformed.

## Results

### Behavioral Data

**FIG. 2:**
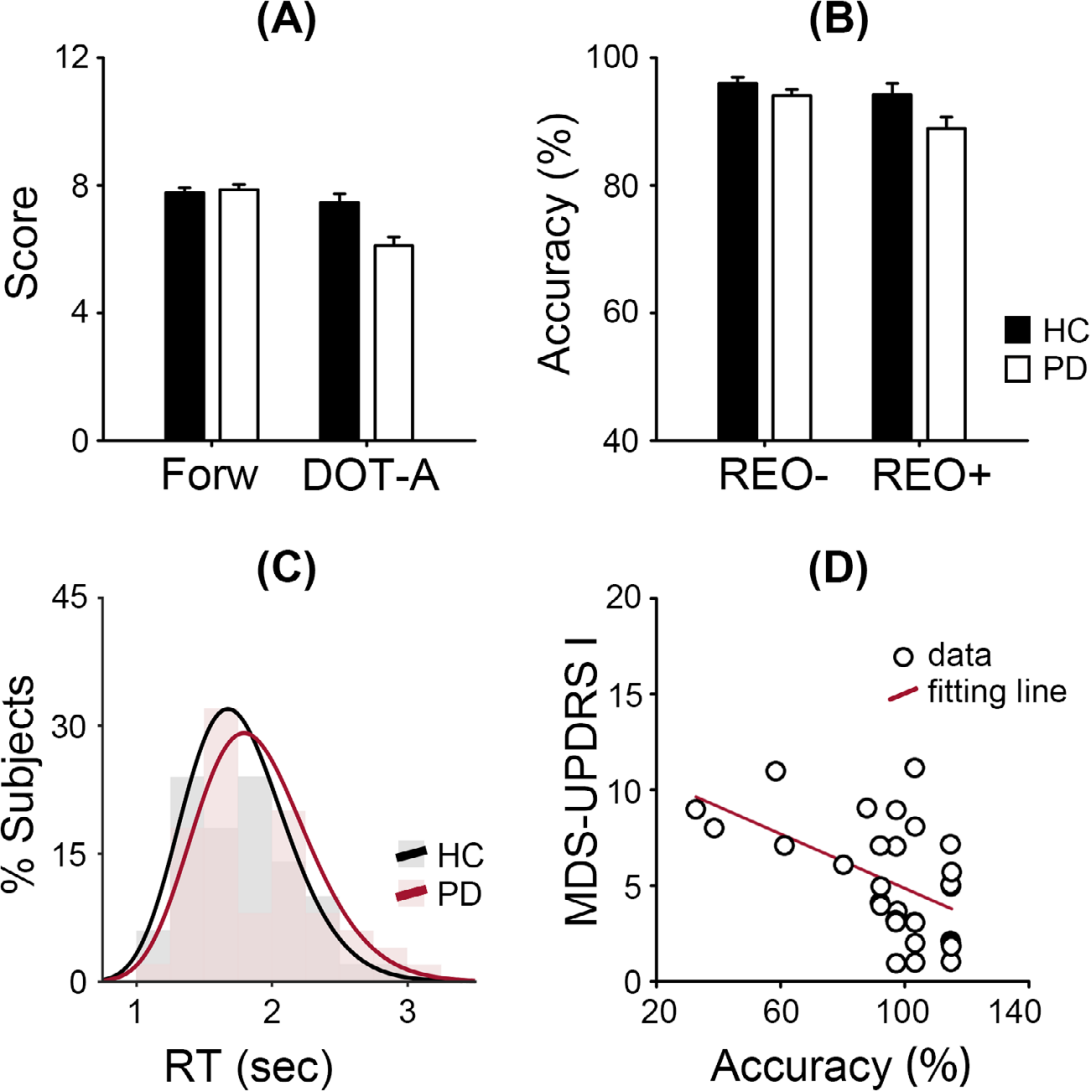
Behavioral data in patients with Parkinson's disease (PD) and healthy controls (HC). (**A**) Mean scores and standard errors of the Adaptive Digit Ordering Test (DOT‐A) and Digit Span Forward Test (Forw). (**B**) Mean accuracy and standard errors of the computerized digit ordering task for “pure recall & without reorder” (REO−) and “reorder & recall” trials (REO+). (**C**) Histogram of reaction times (RT) in “reorder & recall” trials with γ distribution fits. (**D**) The “reorder & recall” accuracy (arcsine transformed) was negatively correlated with the severity of nonmotor symptoms (Movement Disorder Society–sponsored revision of Unified Parkinson's Disease Rating Scale Part I [MDS‐UPDRS I] score). [Color figure can be viewed at wileyonlinelibrary.com]

Figure 2 presents the behavioral data of the computerized digit ordering task and the 2 neuropsychological working memory tests. First, we replicated previous findings that patients with PD scored lower than healthy controls in the Adaptive Digit Ordering Test, but not in the Digit Span Forward Test (Fig. 2A and Table 1). Second, we observed a similar pattern in the accuracy of the digit ordering task (Fig. 2B) with main effects of trial type (*F*_1,98_ = 16.02, *P* < 0.001, η^2^ = 0.14) and group (*F*_1,98_ = 5.27, *P* = 0.024, η^2^ = 0.05), and an interaction between group and trial type (*F*_1,98_ = 3.93, *P* = 0.05, η^2^ = 0.04). Participants were, in general, less accurate in “reorder & recall” than “pure recall” trials. Patients with PD were less accurate than healthy controls, especially in “reorder & recall” trials. However, we found no group difference in reaction time (Fig. 2C). Despite their motor symptoms, patients with PD were as fast as healthy controls. Third, we observed a negative correlation between individual patients' “reorder & recall” accuracy and their severity of nonmotor symptoms (MDS‐UPDRS Part I score, *r* = −0.53, *P* = 0.002; Fig. 2D), when the severity of motor symptoms (MDS‐UPDRS Part III score) was controlled. Patients with a lower task accuracy tended to report more severe nonmotor problems in daily living. The MDS‐UPDRS Part III score itself did not correlate with task performance.

### Replication of Ordering‐Related Regional Activation and Deactivation

**FIG. 3:**
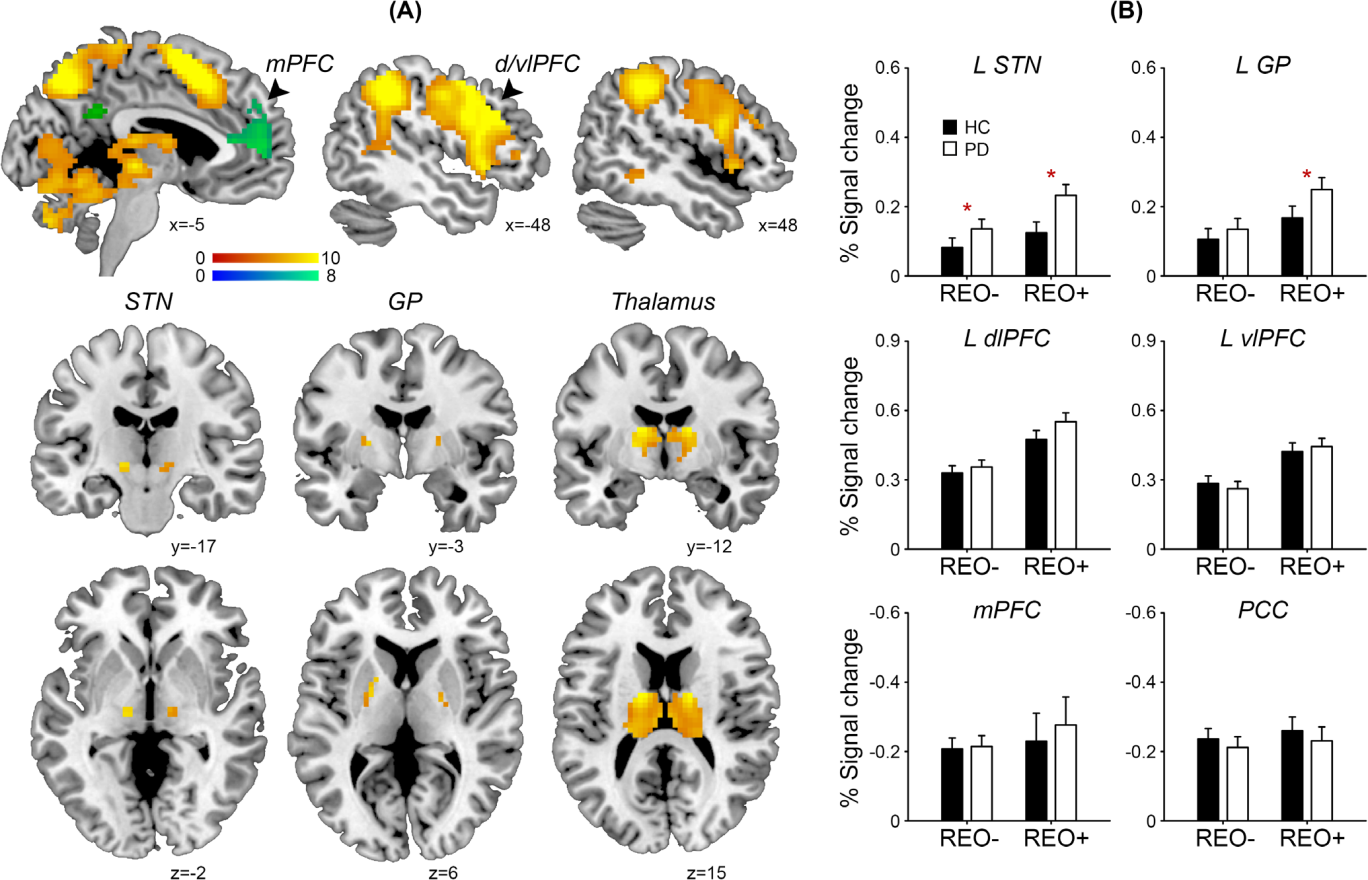
Group differences in regional activation. (**A**) Ordering‐related regional activation (warm colors) and deactivation (cool colors). Color scales indicate *t* values. Coordinates are in Montreal Neurological Institute and Hospital space. (**B**) Means and standard errors of the percent signal change in the regions of interest for “pure recall” (REO−) and “reorder & recall” trials (REO+) in patients with Parkinson's disease (PD) and healthy controls (HC). Asterisks indicate significant group differences (*P* < 0.05). L, left; mPFC, medial prefrontal cortex; d/vlPFC, dorsolateral/ventrolateral prefrontal cortex; PCC, posterior cingulate cortex; STN, subthalamic nucleus; GP, globus pallidus. [Color figure can be viewed at wileyonlinelibrary.com]

We replicated the ordering‐related regional activation and deactivation across groups (Fig. 3A).^11^ Regional activations were greater for “reorder & recall” than “pure recall” trials (whole‐brain 2‐sample *t* test, voxel‐level *P* < 0.05 family‐wise‐error corrected) in the dorsomedial prefrontal cortex (BA8/6: peak in Montreal Neurological Institute and Hospital coordinate system [−6, 15, 51], *t* = 15.66, 1687 voxels), dorsolateral prefrontal cortex (BA46/9: left [−45, 6, 30], *t* = 12.55, 255 voxels; right [39, 33, 33], *t* = 9.73, 245 voxels), ventrolateral prefrontal cortex (BA44/45: left [−45, 6, 27], *t* = 12.37, 172 voxels; right [54, 12, 18], *t* = 9.69, 116 voxels), posterior parietal cortex (BA7/40: left [−27, −69, 36], *t* = 16.27, 2042 voxels), STN (left [−15, −18, −3], *t* = 7.40, 6 voxels; right [12, −15, −3], *t* = 6.64, 8 voxels), external globus pallidus (left [−21, −3, 6], *t* = 8,22, 14 voxels; right [21, −6, 6], *t* = 6.92, 7 voxels), and thalamus (left [−15, −6, 15], *t* = 10.39, 360 voxels; right [15, −6, 15], *t* = 9.94, 250 voxels). Regional deactivations were greater for “pure recall” than “reorder & recall” trials in the default mode network, including the medial prefrontal cortex ([6, 51, 15], *t* = 8.64, 347 voxels) and posterior cingulate cortex ([0, −48, 30], *t* = 7.10, 55 voxels).

### Group Differences in the Subthalamic Nucleus and Globus Pallidus Activation

We observed group differences in the regional activation of the left STN and globus pallidus, but not other regions of interest (Fig. 3B). Note, we focused on the left regions because the ordering‐related regional activation was left‐lateralized in the independent fMRI data set.^11^ In the left STN, we observed a main effect of group (*F*_1,88_ = 3.94, *P* = 0.05, η^2^ = 0.04), an interaction between group and trial type (*F*_1,88_ = 6.80, *P* = 0.01, η^2^ = 0.07), and a main effect of trial type (*F*_1,88_ = 44.23, *P* < 0.001, η^2^ = 0.33). Patients with PD showed greater STN activation than healthy controls, especially in “reorder & recall” trials. In the left globus pallidus, there was an interaction between group and trial type (*F*_1,88_ = 5.38, *P* = 0.02, η^2^ = 0.06) and a main effect of trial type (*F*_1,88_ = 57.41, *P* < 0.001, η^2^ = 0.40), but no main effect of group (*F*_1,88_ = 1.57, *P* = 0.21). Patients with PD showed greater globus pallidus activation than healthy controls in “reorder & recall” trials, but not in “pure recall” trials.

No other regions of interest showed a main effect of group or interaction between group and trial type. We exploratorily analyzed the right STN and globus pallidus and observed similar overactivations in PD.

### Group Differences in STN Functional Connectivity

**FIG. 4:**
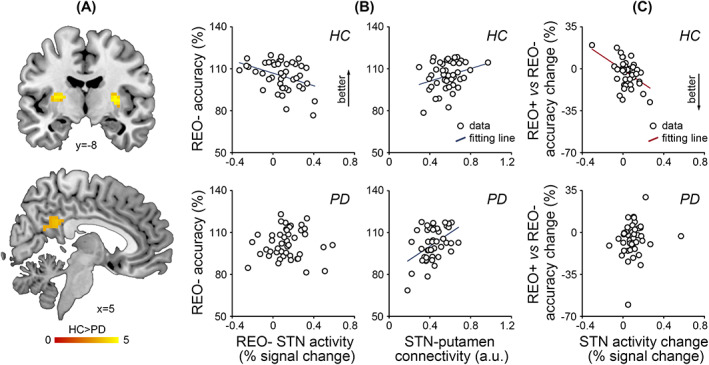
Group differences in functional connectivity and brain–behavior relationships. (**A**) The subthalamic nucleus (STN) functional connectivity with the putamen (top) and posterior cingulate cortex (bottom) was stronger for healthy controls (HC) than patients with Parkinson's disease (PD) (HC > PD). Color scale indicates *t* values. Coordinates are in Montreal Neurological Institute and Hospital space. (**B**) In healthy controls, the accuracy in “pure recall” trials (REO−) was predicted by the STN activation and STN–putamen functional connectivity. In patients, the “pure recall” accuracy was only predicted by the STN–putamen functional connectivity. (**C**) The accuracy difference between “reorder & recall” and “pure recall” trials (REO+ vs REO−) was predicted by the STN activation change in healthy controls, but not in patients. [Color figure can be viewed at wileyonlinelibrary.com]

Having observed group differences in the regional activation of the STN and globus pallidus, we next sought group differences in their functional connectivity (whole‐brain 2‐sample *t* test, cluster‐level *P* < 0.05 family‐wise‐error corrected). We observed stronger time‐course correlations between the left STN and putamen (left [−27, −15, 9], *t* = 5.74, 41 voxels; right [30, −9, 6], *t* = 6.06, 54 voxels) and between the left STN and posterior cingulate cortex ([9, −42, 30], *t* = 3.92, 63 voxels) for healthy controls than patients with PD (Fig. 4A).

Neither the globus pallidus nor prefrontal seeds showed group differences in functional connectivity. No seeds showed group differences in psychophysiological interaction.

### Predicting Task Accuracy with the STN Activation and Functional Connectivity

Patients with PD and healthy controls showed different relationships between task accuracy and STN activation/functional connectivity. In healthy controls (Fig. 4B,C, top), the linear regression models were significant for both “pure recall” accuracy (*F*_3,44_ = 3.57, *P* = 0.02) and ordering‐related accuracy change (“reorder & recall” vs “pure recall,” *F*_3,44_ = 5.24, *P* = 0.004). The “pure recall” accuracy was predicted by the “pure recall” STN activation (*t* = −2.63, *P* = 0.01) and marginally by the STN functional connectivity with the putamen (*t* = 1.92, *P* = 0.06), but not by the STN functional connectivity with the posterior cingulate cortex (*t* < 1). Moreover, the ordering‐related accuracy change was predicted by the STN activation change (*t* = −3.58, *P* = 0.001), but not by the STN functional connectivity with the putamen (*t* < 1) or posterior cingulate cortex (*t* = −1.53, *P* = 0.13). Healthy controls with lower “pure recall” STN activation, greater ordering‐related STN activation change, and stronger STN–putamen functional connectivity tended to perform better (higher “pure recall” accuracy and smaller ordering‐related accuracy change).

In patients with PD (Fig. 4B,C, bottom), the linear regression model was significant for the “pure recall” accuracy (*F*_3,44_ = 4.46, *P* = 0.008), but not for the ordering‐related accuracy change (*F* < 1). The “pure recall” accuracy was predicted by the STN functional connectivity with the putamen (*t* = 3.34, *P* = 0.002), but not by the “pure recall” STN activation (*t* < 1) or the STN functional connectivity with the posterior cingulate cortex (*t* = −1.29, *P* = 0.21). Patients with PD with stronger STN–putamen functional connectivity tended to be more accurate in “pure recall” trials. No variables of interest predicted the ordering‐related accuracy change in the patients.

## Discussion

Deficits in sequential working memory may lead to everyday difficulties in planning (eg, What to do first?) and language processing (eg, What happened first? What to say first?). In this study, we demonstrated neural markers of the deficits in patients with de novo PD with mild clinical symptoms. First, we confirmed that patients with PD were less accurate than healthy controls in recalling digit sequences, especially when they had to reorder the digits to generate a new sequence (“reorder & recall”). The deficits were related to the presence of nonmotor problems in daily living, as quantified by the MDS‐UPDRS Part I subscale. Second, we observed PD‐related changes in the regional activation and functional connectivity of the left STN. In patients with PD, the STN was overactivated, especially for “reorder & recall” trials and lost synchrony with the putamen (weaker correlation between physiological signals). More important, the modulatory role of the STN was significantly weakened in patients with PD. In healthy controls, individual participants' task accuracy can be predicted by the regional activation and functional connectivity of the STN. Healthy participants who showed lower “pure recall” STN activation and stronger STN–putamen functional connectivity tended to perform more accurately in maintaining sequential information. Healthy participants who showed greater STN activation change had smaller accuracy costs in manipulating sequential information (“reorder & recall” vs “pure recall”). In patients with PD, however, the “pure recall” accuracy was only predicted by the STN–putamen functional connectivity. The contribution of the STN activation or activation change was absent, probably as a result of a ceiling effect in PD (eg, lack of variability). We did not observe a significant change in the lateral prefrontal cortex. Aging and disease may not influence the neural processes of sequential working memory in the same manner. Although the lateral prefrontal cortex exhibited age‐related alteration in regional activation and interregional interaction,^11^ it might not specifically contribute to the deficits in de novo PD.

The STN is an essential modulator of basal ganglia loops. It receives projections from the brainstem (including noradrenergic projections from the locus coeruleus and dopaminergic projections from the substantia nigra pars compacta), thalamus, external globus pallidus, and frontal cortex and projects back to the internal and external globus pallidus, striatum, and brainstem.^25^, ^26^ The observed STN dysfunction may result from the early affection of the locus coeruleus and substantia nigra pars compact in pathological stages 2 to 3 rather than direct damages to the prefrontal cortex in pathological stages 5 to 6.

STN has been associated with working memory. Some researchers found that patients with PD with STN deep brain stimulation responded faster and more accurately in visuospatial and emotional working memory tasks when the stimulation was switched ON versus OFF.^27^, ^28^, ^29^ Other researchers observed the opposite: patients with PD made more errors and slower responses in visuospatial and verbal working memory tasks when the stimulation was switched ON versus OFF.^30^, ^31^ These studies revealed mixed results, probably because they looked at more advanced stages of PD when the cascade of α‐synuclein pathology and the chronic effect of the medication lead to a more complicated situation. This study is an initial attempt to link the STN with sequential working memory in the early stages of PD. We proposed that the STN is involved in maintaining and manipulating sequences online and that the dysfunction of the STN contributes to sequential working memory deficits in de novo PD.

The temporary maintenance of sequences is often assumed to use a competitive queuing mechanism that comprises a parallel planning layer and a competitive choice layer.^32^, ^33^, ^34^ The nodes in the parallel planning layer represent items in a to‐be‐recalled sequence. The order of the items is represented in terms of a primacy gradient of node activation. Namely, the node activation of the first item is strongest, and the node activations of the subsequent items decline monotonically toward the last item. The nodes in the competitive choice layers are excited by corresponding nodes in the parallel planning layer and inhibited by competitive nodes in the same layer. Recalling a sequence is realized via iterative processes. At each iteration, the most active node in the competitive choice layer is selected, and the corresponding node in the parallel planning layer is suppressed by the feedback projection from the competitive choice layer. At the next iteration, the second strongest node becomes the most active. The competitive queuing mechanisms are thought to reside in the prefrontal cortex, a notion supported by electrophysiological evidence from macaques^9^, ^35^, ^36^, ^37^ and magnetoencephalographic evidence from humans.^38^ However, the direct evidence regarding the contribution of the basal ganglia is mostly missing.

The flexible manipulation of sequences is even less understood. Recently we proposed that rearranging sequential items may require a dynamic adjustment of node activations in the parallel planning layer, for example, inhibiting items that should be moved downward and enhancing items that should be moved upward in the new order.^11^ The adjustment may be supported by a basal ganglia gating mechanism similar to that proposed for action selection. In Frank's model, the striatum modulates the execution of a particular action, whereas the STN modulates the decision threshold and reduces premature responding.^39^ In other models, the STN supports the suppression of alternative competing actions when one action is selected.^40^ However, the real picture may be more complicated than the computational models have anticipated given the afferent and efferent projections of the STN. To further understand its role (and that of other basal ganglia structures) in sequential working memory, perioperative intracranial electrophysiological recordings from the STN in PD might be helpful.^41^

In conclusion, we demonstrated the effect of PD on the neural system for sequential working memory in de novo patients with mild clinical symptoms. The neural system comprises the lateral prefrontal cortex, posterior parietal cortex, STN, globus pallidus, and thalamus. The STN plays a modulatory role in maintaining and manipulating sequences in healthy adults. Healthy participants who showed lower “pure recall” STN activation, greater ordering‐related STN activation change, and stronger STN–putamen functional connectivity tended to perform better. In patients with PD, the STN was overactivated and lost synchrony with the putamen. Thus, the modulatory role of the STN was weakened mainly. Individual patients' performance correlated with the functional connectivity, but not the regional activation or activation change of the STN. It implies that downregulating STN activation and upregulating STN functional connectivity may be a potential strategy for enhancing sequential working memory in PD.

## Author Roles

(1) Research Project: A. Conception, B. Organization, C. Execution; (2) Statistical Analysis: A. Design, B. Execution, C. Review and Critique; (3) Manuscript: A. Writing of the First Draft, B. Review and Critique.

Z.Y.: 1A, 1B, 1C, 2A, 2B, 3A

G.Z.: 1C, 2C, 3B

Y.Z.: 1B, 1C, 3B

S.L.: 1C, 3B

N.L.: 1C, 3B

X.Z.: 1C, 3B

W.X.: 1C, 3B

T.F.M.: 1A, 2C, 3A

## Full financial disclosures for the previous 12 months

The authors have nothing to report.
